# The activity of indigo carmine against bacteriophages: an edible antiphage agent

**DOI:** 10.1007/s00253-025-13414-4

**Published:** 2025-01-25

**Authors:** Sada Raza, Bartłomiej Bończak, Nataliia Atamas, Aneta Karpińska, Tomasz Ratajczyk, Marcin Łoś, Robert Hołyst, Jan Paczesny

**Affiliations:** 1 Institute of Physical Chemistry, Polish Academy of Sciences, Kasprzaka 44/52, 01-224 Warsaw, Poland; 2 Taras Shevchenko National University of Kyiv, Hlushkova Avenue 4, Kiev, 03127 Ukraine; 3 Department of Molecular Genetics of Bacteria, Faculty of Biology, University of Gdańsk, Wita Stwosza 59, 80-308 Gdańsk, Poland; 4 Phage Consultants, Partyzantów 10/18, 80-254, Gdańsk, Poland

**Keywords:** Bacteriophages, Antiphage agents, Indigo carmine, Bacteria-based industries, Antivirals

## Abstract

**Abstract:**

Bacteriophage infections in bacterial cultures pose a significant challenge to industrial bioprocesses, necessitating the development of innovative antiphage solutions. This study explores the antiphage potential of indigo carmine (IC), a common FDA-approved food additive. IC demonstrated selective inactivation of DNA phages (P001, T4, T1, T7, λ) with the EC_50_ values ranging from 0.105 to 0.006 mg/mL while showing no activity against the RNA phage MS2. Fluorescence correlation spectroscopy (FCS) revealed that IC selectively binds to dsDNA, demonstrated by a significant reduction in the diffusion coefficient, whereas no binding was observed with ssDNA or RNA. Mechanistically, IC permeates the phage capsid, leading to genome ejection and capsid deformation, as confirmed by TEM imaging. Under optimal conditions (50 °C, 220 rpm), IC achieved up to a 7-log reduction in phage titer, with kinetic theory supporting the enhanced collision frequency induced by agitation. Additionally, IC protected *E. coli* cultures from phage-induced lysis without affecting bacterial growth or protein production, as demonstrated by GFP expression assays. IC’s effectiveness and environmental safety, combined with its FDA approval and cost-effectiveness, make it a promising antiphage agent for industrial applications.

**Key points:**

• *Indigo carmine effectively inactivates a broad spectrum of bacteriophages, offering protection to bacteria in industrial cultures.*

• *A novel application of indigo carmine as a food-grade, environmentally safe, and FDA-approved antiphage agent protecting bacterial cultures.*

• *Antiphage activity arises from indigo carmine’s interaction with DNA within the phage capsid without harming bacterial cells or compromising protein production in bacterial cultures.*

**Graphical abstract:**

**Figure:**
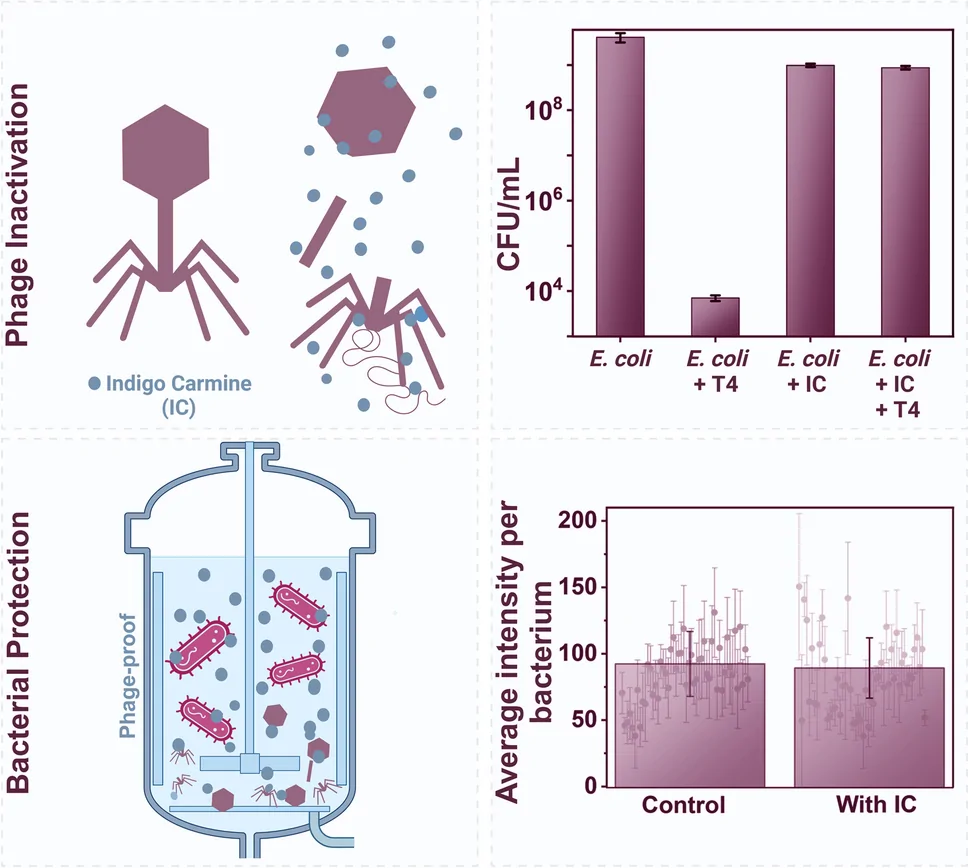

**Supplementary Information:**

The online version contains supplementary material available at 10.1007/s00253-025-13414-4.

## Introduction

Advancements in molecular biology and biotechnology have unlocked the potential of harnessing microorganisms’ metabolic processes for industrial applications. With a better understanding of bacteria and yeast, microbes are now used for the production of food (e.g., yogurt and cheese (Johnson et al. 2021); fermentation of vegetables like cabbage (Chen et al. 2020)); manufacture of biofertilizers (Nosheen et al. 2021), drugs, including insulin (Govender et al. 2020); and the treatment of diseases (like cystic fibrosis, hemophilia, and hepatitis B) to name a few. Sales of microbial-origin pharmaceuticals now exceed 13 billion US dollars annually (*Microbial Products Market Size and Growth 2023 to 2032*, 2023). Moreover, gut microbiome research has highlighted the critical role of the symbiotic relationship between bacteria and humans (Wibowo et al. 2021). In all of these cases, bacteria are our allies.

But these allies have an enemy—bacteriophages, i.e., viruses that infect bacteria. Bacteriophage (phage) infections in the production environment lead to the spread of persistent contaminations, followed by losses of the bioproduct (Zahn & Halter 2020). Moreover, this effect is worse when working on a large scale, such as in biofoundries, where the reappearance rate of phages is highly common in biotech plants (Łos et al. 2004; Marton et al. 2023). For instance, it was shown that raw milk is the most common source of new phages in the dairy industry (Calendar and Abedon 2006; Połaska & Sokołowska 2019). This is in line with the study by del Rio, where phages were detected in 37% of raw milk samples (del Rio et al. 2007). The dairy industry loses from 1 to 10% of batches due to phage infection (Calendar and Abedon 2006).

The primary efforts are aimed at reducing the probability of phage infections, both at the design level (by optimizing protocols for wastes and handling of raw materials, biofoundry and process layout, and personnel training) and by employing microbiological strategies (periodic changes of bacterial strains, cocktails of bacterial strains and development of phage-resistant mutants) (Samson & Moineau 2013; Sturino & Klaenhammer 2006). Regrettably, only a handful of solutions are currently available to mitigate the impact of bacteriophage infections when they occur (Guglielmotti et al. 2012; Raza et al. 2023).

Phage infections necessitate proper disinfection of facilities. Even a low number of survivor phages can cause recurring infections (Łoś, 2020). It is important to note that some phages can resist extreme conditions, such as being heated at 90 °C for 15 min or, in some cases, even boiling (Guglielmotti et al. 2012). Several physical factors, such as temperature, the acidity of the environment, UVC irradiation or salinity, and ions, were individually explored to determine the survivability of bacteriophages under harsh conditions (Jończyk et al. 2011; Ma et al. 2021; Wdowiak et al. 2022). It was also found that phages are highly resilient against most physical factors. Moreover, radiation-based methods face significant difficulties due to hindered penetration into cloudy media or tight spaces within the biofoundries (Atamer et al. 2013). Another means of fighting phage infections is the utilization of specific chemical agents. For instance, Virkon, which contains potassium monopersulfate as its primary active ingredient, has strong antiphage and bactericidal properties (Morin et al. 2015). However, these compounds are usually very toxic to humans and the environment and might cause corrosion on surfaces and equipment damage. Another effective method for inactivating phages is the use of quaternary ammonium compounds (QACs), which are active ingredients in over 200 disinfectants recommended by the U.S. EPA for inactivating SARS-CoV-2 (Hora et al. 2020).

Among the new research areas on antiphagents (i.e., anti-bacteriophage agents), little has been done to develop new technologies that aim at deactivating phages inside an operating bioreactor, such as antiphage nanoparticles (Richter et al. 2022; Richter et al. 2021a, b). Therefore, it is a struggle to propose a method for the inactivation of bacteriophages that is safe for bacteria, humans, and the environment, relatively inexpensive (as opposed to gold nanoparticles Richter et al. 2021b), and easily implemented. Advancements in molecular biology and biotechnology have unlocked the potential of microorganisms for various industrial applications, from food production to pharmaceuticals. However, bacteriophage infections remain a persistent challenge, jeopardizing bacterial cultures and causing significant economic losses. Current methods to combat phages, such as toxic disinfectants or expensive nanoparticles, often have limitations, including environmental risks, high costs, or adverse effects on bacterial viability and productivity.

Here, we propose a solution that meets these requirements. This study presents a new method for inactivating bacteriophages using an agent frequently used in the food industry, cosmetics, fabric staining, and medicine—indigo carmine (IC), also known as E132. Indigo is confirmed to have anti-inflammatory, antioxidant, and antiviral properties (Qi-yue et al. 2020; Tu et al. 2020). Experiments with extracts of *Isatis tinctora* against the Japanese encephalitis virus (JEV) display the potential virucidal properties of the herb and its ability to inhibit viral attachment (Chang et al. 2012). Lignans extracted from the root of this herb have also shown some anti-influenza properties (Yang et al. 2013). Inhibition of viral replication can also be achieved by a 3,2′-bisindole isomer of indigo-indirubin (Chan et al. 2018). Due to the COVID-19 pandemic, the antiviral activity of numerous herbs and natural compounds has been revisited (Fuzimoto & Isidoro 2020). Experiments performed with *Indigo naturalis* tone down the detrimental effects of coronavirus on the lungs (Tu et al. 2020).

All of these studies, as well as other reports on the antiviral properties of natural compounds, target the metabolism of host cells to block the virus cycle at various stages (De Clercq & Li 2016). Therefore, antiviral drugs designed against eukaryotic viruses are often ineffective against phages. Prokaryotic cells use different enzymatic and synthetic pathways. It is impossible to directly relate the antiviral to the antiphage activity of active compounds.

Here, we demonstrate that indigo carmine acts directly against virions, causing their inactivation instead of interfering with the replication cycle inside bacteria. The main aim of the research is to explore the possible application of indigo carmine directly in bioreactors to protect bacteria from phages. IC appears safe for both bacteria and humans. Thus, using IC protects the biofoundries and does not spoil the final product. The present study introduces indigo carmine (IC) as a novel antiphage agent in this context. IC stands out because it is FDA-approved, cost-effective, and safe for both bacteria and humans. Unlike existing chemical agents, IC selectively targets DNA phages without harming bacterial cultures or interfering with protein expression, making it highly compatible with industrial processes. Furthermore, this study elucidates the mechanism of IC’s activity, highlighting its interaction with phage DNA and the conditions that enhance its efficacy, such as temperature and agitation.

## Materials and methods

### Materials and chemicals

Indigo carmine (E132) was purchased from Food Colors, Poland, as a blue food colorant and used as received. The composition of solid LB agar, used to prepare plates for microbiological tests, was as follows: 15 g/L of agar, 10 g/L of NaCl, 10 g/L of tryptone, and 5 g/L of yeast extract. Liquid LB medium was used to prepare overnight and refreshed cultures, which had identical compositions except for the absence of agar. Both LB agar and LB medium were directly ordered from Carl Roth (Germany). TM buffer, set at 7.4 pH, was prepared by mixing Tris base (484.56 mg, 10 mM concentration in the final solution), CaCl_2_ (800 μL, 5 μM), MgSO_4_ (481.66 mg, 10 mM concentration in the final solution), and 400 mL deionized water. All the media were autoclaved before use.

### Consumables

Fifty millilitters of sterile falcon tubes (NeoCulture centrifuge tubes—made of PP, 50 mL, self-standing, sterile) and phage-safe 1.5-mL Eppendorf-type tubes (B-1429 and B-2278) (Richter et al. 2021a, b) were purchased from Bionovo (Poland).

### Bacteriophages

T1 (*E. coli* BL21), T4 (*E. coli* BL21), T7 (*E. coli* BL21), MS2 (*E. coli* C3000), and λ (*E. coli* Top10) bacteriophages were purchased from Phage Consultants (Poznan, Poland). P001 (*Lactococcus lactis*) phages were purchased from DSMZ (Germany). The stock suspensions’ titers ranged from 10^11^ to 10^12^ PFU/mL, which was later adequately diluted for the experiments. The initial titers of the phages used in the experiments were T4 (4 × 10^5^ PFU/mL), T7 (6 × 10^4^ PFU/mL), T1 (4 × 10^3^ PFU/mL), λ (9 × 10^6^ PFU/mL), P001 (4 × 10^5^ PFU/mL), and MS2 (10^9^ PFU/mL).

### Bacteria

A single colony of *E. coli* (the required strain) was picked up from the stock plate and transferred to 10 mL of LB medium to prepare the bacterial cultures. This sample was then incubated at 37 °C in the shaker (Orbital Shaker-Incubator ES-20, 220 rpm). The overnight culture was refreshed by mixing 2.5 mL with 7.5 mL of fresh LB medium and incubating at 37 °C for approximately 1 h to obtain the desired OD_600_ (around 1).

### Double overlay titration for phage analysis

Petri plates were first filled with 20 mL of LB agar medium and left to solidify. 4 mL of top LB agar (prepared with liquid medium and 0.5% agar instead of 1.5% agar) was then mixed with 200 μL of the refreshed culture of *E. coli* (the appropriate strain) and poured onto the plate. Appropriate dilutions of the phage solution were prepared, and from each dilution, 7 to 8 droplets of 5 μL solution were placed onto the top agar layer. The number of plaques was counted after incubation of the plates at 37 °C for 24 h. In the case of λ, the plates were incubated at 45 °C to initiate the lytic cycle. The experiments were performed in three biological replicates. Student’s *t*-test was performed to evaluate whether observed differences were statistically significant (*P < 0.05; **P < 0.01; ***P < 0.001).

### Indigo carmine: time and temperature

The effect of IC on T4, T1, T7, λ, MS2, and P001 phages was considered. An hourly analysis was carried out with indigo carmine. The incubation was first performed on T4 at a range of temperatures to establish the conditions required to achieve complete inactivation. The chosen range comprised 35 °C, 45 °C, 50 °C, and 55 °C. All the other phages were then incubated at the chosen temperature, i.e., 50 °C.

### Indigo carmine: dose–response tests

Phages were incubated in TM buffer with various concentrations of indigo carmine at 37 °C for 24 h. As a control, samples with phages without any IC added were tested. The number of phages was estimated via the plaque count method described in the previous section. All experiments were performed in triplicates. The experiments were performed in three biological replicates. Student’s *t*-test was performed to evaluate whether observed differences were statistically significant (*P < 0.05; **P < 0.01; ***P < 0.001).

### E. coli in the presence of IC and T4

This experiment was carried out in triplicates in the orbital shaker using *E. coli* BL21. Four flasks containing only *E. coli*, *E. coli* and T4, *E. coli* and IC, and *E. coli*, IC, and T4, respectively, were taken. The samples were left inside the incubator for 6 h at 37 °C. After the incubation, the number of bacterial colonies in each sample was computed using the standard plate count method. The experiments were performed in three biological replicates. Student’s *t*-test was performed to evaluate whether observed differences were statistically significant (*P < 0.05; **P < 0.01; ***P < 0.001).

### Induction of GFP production

To assess the effect of IC on the functioning of bacterial cells, overnight cultures of GFP *E. coli* were prepared with and without IC. The selected strain of *E. coli* harbored a plasmid coding green fluorescence protein (GFP) and showed resistance to chloramphenicol and kanamycin. Bacteria were prepared according to the standard procedures., i.e., 25 mg of chloramphenicol and 50 mg of kanamycin were added to 1 L of LB media. Isopropyl β-D-1-thiogalactopyranoside (IPTG) was added to LB media to a final concentration of 1 mM to induce the production of the GFP protein. A single colony from the agar plate was inoculated into LB medium for overnight culturing (37 °C, 200 rpm). These samples were refreshed according to the protocol described above and analyzed under a fluorescence microscope (Nikon Eclipse 50i).

### Fluorescence correlation spectroscopy (FCS)

FCS is a technique that measures the diffusion coefficients of individual fluorescent molecules in a sample. Mathematical analysis of fluorescence fluctuations allows for determining the number of mobile particles in the focal volume and their diffusion coefficients. Measurements were performed using a Nikon Eclipse TE2000U confocal microscope with the FCS PicoHarp 300 set. The fluorescence was excited using a 488 nm pulse laser. Observations were carried out using a × 60 lens (NA 1.2) with water immersion. The temperature (25 ± 0.5 °C) was maintained using a climate chamber (Okolab, Italy). Each measurement was preceded by calibration in the same medium as the tested sample. More details on the probes and data fitting can be found in the Supporting Information.

### Transmission electron microscopy (TEM)

TEM study was conducted using an electron microscope Tecnai Spirit BioTWIN with a digital camera. The samples were prepared for negative staining by placement on C400 Cu100 grids (400 Mesh, copper, and carbon layer coating) manufactured by EM Resolutions. Samples were negatively stained with 2% uranyl acetate in water at room temperature for 15 s.

## Results

### The effect of IC on T4 at different temperatures

**Fig. 1:**
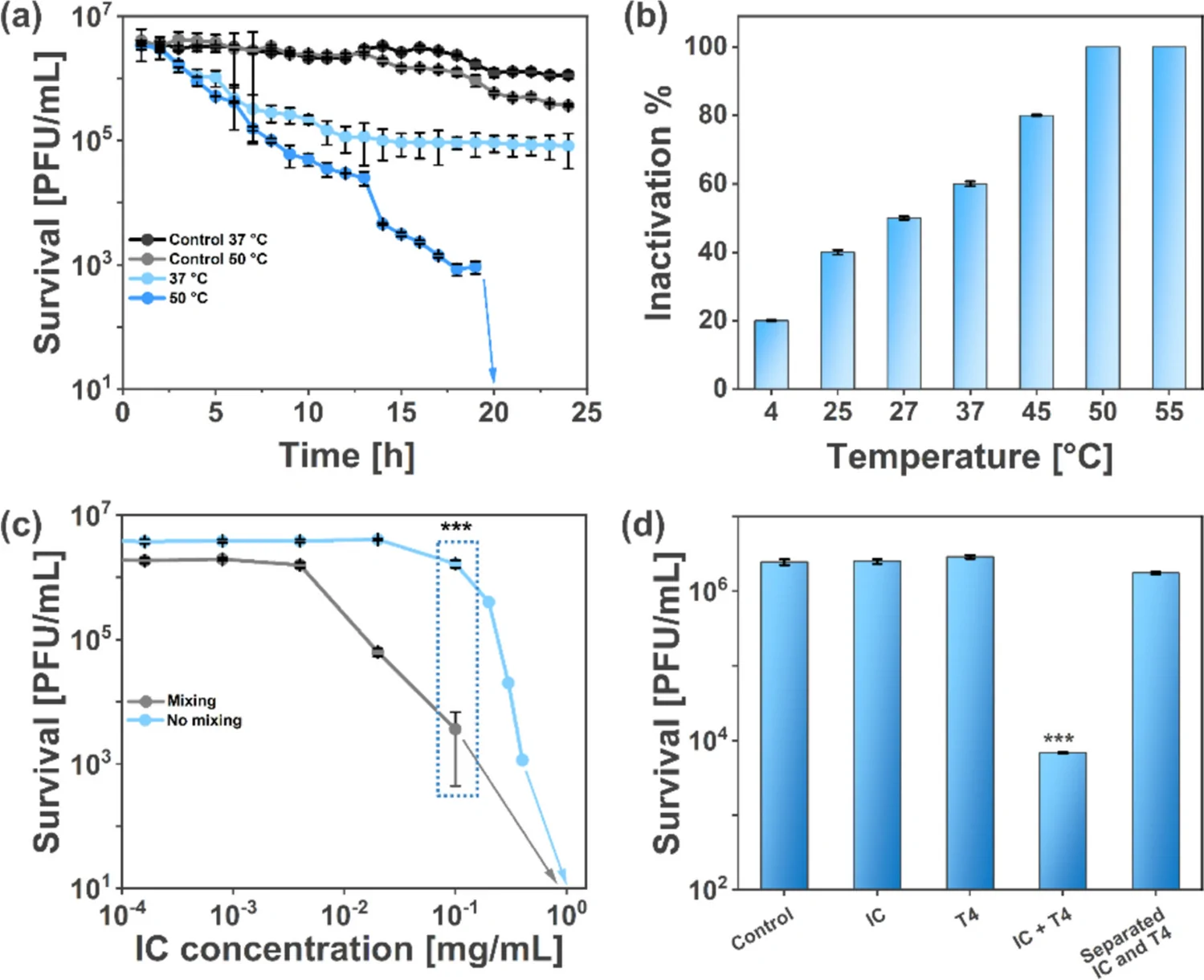
**a** Analysis of T4 titer at 37 °C and 50 °C (no mixing) in the presence and absence of indigo carmine. The concentration of IC at 37 °C was 2 mg/mL and 0.5 mg/mL at 50 °C. 0.5 mg/mL of IC caused no effect at 37 °C without mixing. The arrow marks the drop in the titer below the detection limit. **b** Experimental efficiency of IC against T4 bacteriophage at temperatures varying from 4 to 55 °C. The phage samples were incubated with IC for 24 h before performing titration. **c** Effect of mixing (220 rpm) on antiphage activity of indigo carmine against T4 bacteriophages at 50 °C. The marked *P*-values (stars) correspond to the statistical difference between IC concentrations of 10^−1^ mg/mL. Arrows mark the drop in the titer below the detection limit. **d** The panel aims to establish the root cause of inactivation. Five different samples (left to right) were incubated at 50 °C (no mixing) and titrated as follows: (A) control sample with only phages in TM buffer (no IC), (B) IC only incubation followed by addition of phages just before titration, (C) phage only incubation followed by addition of IC just before titration, (D) incubation of IC and phages together followed by titration, and (E) separate incubation of IC and phages followed by combining them and immediate titration

We selected T4 (Caudoviricetes) because the majority of phages (over 96%) exhibit a similar, that is, tailed morphology (Ackermann 2009). T4 features a capsid (a protein shell containing DNA), a tail, and fibers. It serves as an excellent model for researching antiphagents (agents that inactivate bacteriophages), which is vital in biotechnology, where active compounds are produced in bacteria (Raza et al. 2023). First, we incubated T4 phages with indigo carmine at 2 mg/mL at 37 °C. IC is inexpensive, so it can be utilized at relatively high concentrations. Thirty-seven degrees Celsius is the standard operating temperature for bioreactors. We observed a modest decrease of more than 1 log of the T4 phage titer, from around 2 × 10^6^ to around 9 × 10^4^ PFU/mL. The titer reached a constant value after 10 h of incubation (Fig. 1a). An increase in IC concentration above 2 mg/mL did not change the results (see Supporting Information, Fig. S1).

Next, we studied the influence of temperature on the efficacy of IC. As in previous research on antiphagents (Richter et al. 2021a, b), the activity of IC increased with the increase in temperature. At 50 °C, IC (2 mg/mL) caused a 2 log decrease after around 12 h and complete inactivation (the titer dropped below the detection limit, i.e., more than 6 log) after overnight incubation (versus around 1 log at 37 °C) (Fig. 1a). It should be noted that temperature alone (control samples) caused some decrease in the phage titer, resulting in a final titer (after 24 h) of around 3.65 × 10^5^ at 50 °C versus 2 × 10^6^ PFU/mL at 37 °C.

The bacteriophages were exposed to IC at the specified temperatures (4 to 55 °C) for 24 h before titration (Fig. 1b). This prolonged exposure ensured accurate assessment of IC activity across the temperature range, confirming increased efficacy at higher temperatures, with complete inactivation observed at 50 °C.

We incubated T4 for 24 h at 50 °C with IC ranging from 0.0001 to 1 mg/mL to find the optimal parameters of the process. Around 1 log deactivation was observed at 0.1 mg/mL, whereas complete deactivation required 0.5 mg/mL (Fig. S1). The optimal conditions for IC’s antiphage activity were determined to be a temperature of 50 °C, an IC concentration of at least 0.5 mg/mL, and an initial phage titer not exceeding ~ 10⁷ PFU/mL, ensuring maximum deactivation within a 24-h incubation period.

We aimed to gain insight into the mechanism of IC action. Thus, we compared the titer of phage suspensions in the following samples: (A) only phages incubated for 12 h at 50 °C; (B) IC incubated for 12 h at 50 °C, with phages added only 5 min before the titration (i.e., after 11 h 55 min after the start of the experiment); (C) phages incubated for 12 h at 50 °C, and pristine IC added only 5 min before the titration (i.e., after 11 h 55 min after the start of the experiment); (D) IC and T4 phages were incubated together for 12 h at 50 °C; and (E) IC and T4 phages were both incubated separately for 12 h at 50 °C, then mixed, and titrated. This was done to verify whether the decomposition of IC (Chowdhury et al. 2020) (expected drop in sample (B)) or change of capsid structure at an elevated temperature (Kostelic et al. 2022) (expected drop in sample (C)) had a crucial role in the process. The decrease in phage titer occurred only when IC was incubated with T4 from the beginning of the experiment, i.e., in sample (D) (Fig. 1d).

### The effect of IC on T4 upon mixing

We observed that mechanical agitation enhanced the efficiency of IC. T4 phages were incubated for 24 h with IC (ranging from 0.0001 to 1 mg/mL, Fig. 1c) with (orbital shaker, 220 rpm) and without mixing at 50 °C. A 3-log decline in PFU/mL was noticed at 0.1 mg/mL of IC upon mixing, whereas without mechanical agitation, the effect was only below 1 log (Fig. 1c). A similar 3-log decrease without mechanical agitation required a five times higher concentration of IC (i.e., 0.5 mg/mL). Mixing increases the frequency of collisions. Based on the kinetic theory, the frequency of collision can be estimated based on Eq. 1:

1 $$Z=n\sigma \langle v\rangle$$

Where *n* is the number density of the particles (number of particles per unit volume), σ is the collision cross-section), and ⟨v⟩ is the average relative speed between the particles. Depending on the exact conditions and the location of the virions and IC in the sample (closer to the edge or the center of the vial), the relative velocity increased by a few thousand times. Since *n* and σ do not change, the increase in the relative velocity was directly correlated with the increased number of collisions.

### Evaluation of IC action against T4

**Fig. 2:**
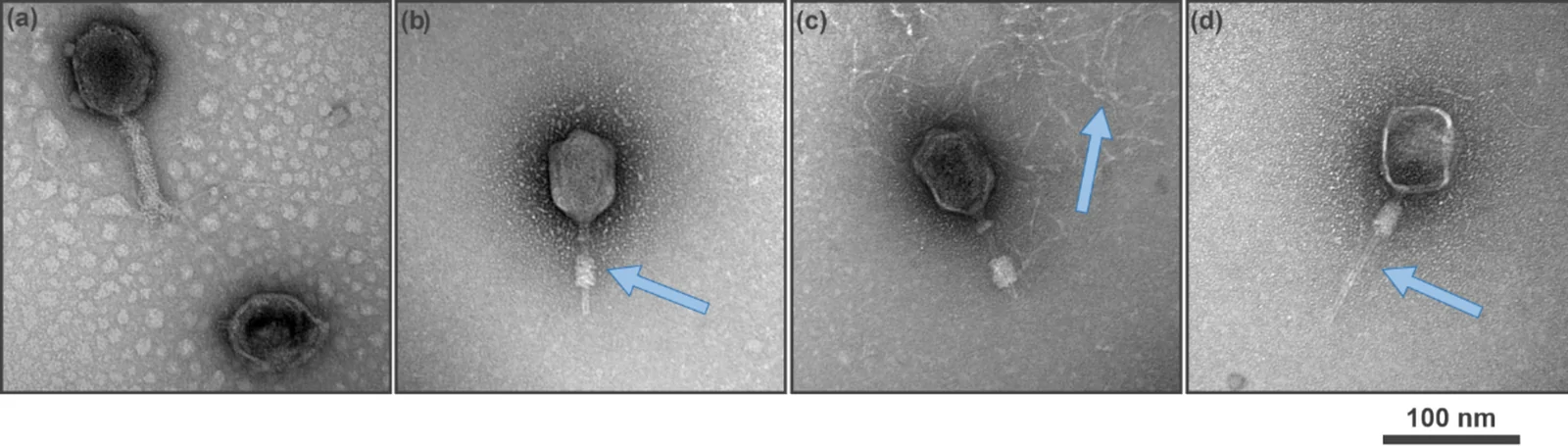
**a**–**d** TEM of T4 bacteriophages in TM buffer (**a**) and in indigo carmine solution (0.5 mg/mL) after incubation at 50 °C for 24 h (**b**). The blue arrows in the images highlight the release of DNA, contracted sheath, and the tube post-tail contraction, respectively. The bar corresponds to 100 nm

We also aimed to gain insight into the mechanism of IC action from the point of view of virions (0.5 mg/mL, 50 °C, 24 h, mixing 220 rpm). We used transmission electron microscopy (TEM) to visualize the differences in T4 virions before (Fig. 2a) and after (Fig. 2b) experiment. First, indigo carmine caused the contraction of the sheath in the vast majority of observed virions. Such a change usually drives the tail tube through the bacterium’s outer membrane upon attachment to the host cell (Leiman et al. 2010). The tail sheath contraction is irreversible. It was also observed that the shape of the capsid was rounded, and the DNA was ejected in some particles. In the absence of indigo carmine, the body of the bacteriophage remained intact, even at an elevated temperature.

### The effect of IC on T1, T7, λ, P001, and MS2

**Fig. 3:**
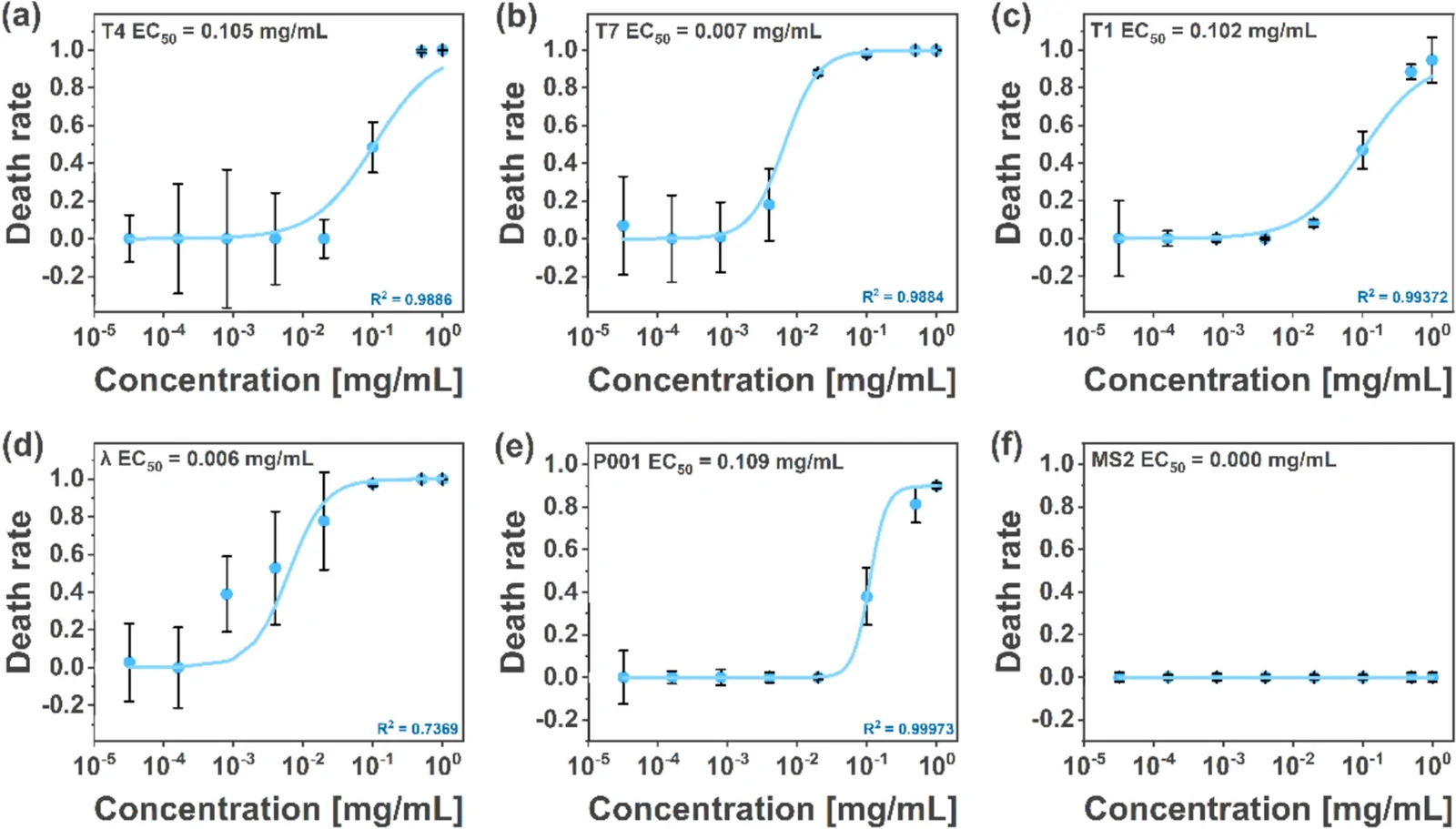
Hills curves showing the effect of indigo carmine (50 °C, 24 h, no mixing) on different phages—T4 (**a**), T7 (**b**), T1 (**c**), λ (**d**), P001 phage (**e**), and MS2 (**f**)

We established IC’s EC_50_ (half-maximal effective concentration) values at 50 °C after 24 h incubation against T4, T1, T7, MS2, λ, and P001 phages (mixing) (Fig. 3). The values of EC_50_ were calculated upon fitting the date to the Hill equation (Prinz 2010). We also normalized the values to account for differences in the initial concentrations of various phages. It was found that recalculated EC_50_ was 4.03 × 10^14^ molecules of indigo carmine per one T4 phage (EC_50_ around 0.105 mg/mL, initial titer around 4 × 10^5 ^PFU/mL), 8.91 × 10^14^ molecules per one T7 phage (approximately 0.007 mg/mL, initial titer around 6 × 10^4 ^PFU/mL), 1.77 × 10^17^ molecules per one T1 phage (around 0.102 mg/mL, initial titer around 4 × 10^3 ^PFU/mL), 4.3 × 10^13^ molecules per one λ phage (around 0.006 mg/mL, initial titer around 9 × 10^6^ PFU/mL), and 2.65 × 10^15^ molecules per one P001 phage (around 0.109 mg/mL, initial titer around 4 × 10^5 ^PFU/mL). Surprisingly, indigo carmine was ineffective against MS2 phages (Fig. 3e). We found that at higher phage titers (above around 10^9 ^PFU/mL), 0.5 mg/mL of IC was insufficient to allow for complete phage deactivation (cf. Fig. S2).

### IC interactions with DNA and RNA

We aimed to explain the ineffectiveness of IC against MS2. We rejected the hypothesis that this was simply caused by the difference in morphology. Other studied bacteriophages belong to a broad range of families or even orders. We hypothesized that the IC activity differed because MS2 was the only tested RNA phage, while all others were DNA phages. It is known that small molecules (e.g., phage staining by dyes (Janczuk et al. 2017)) can penetrate the capsid and bind to the stored genetic material. We aimed to verify if this was true for IC. Thus, we used fluorescence correlation spectroscopy measurements to verify any differences in the binding of IC to RNA and DNA. We traced IC, which shows fluorescence at 528 nm (Zinellu et al. 2010) upon irradiation with a laser of wavelength 488 nm. First, we obtained IC’s diffusion coefficient and the hydrodynamic radius without nucleic acids (Fig. S4). Next, we compared the values obtained upon IC incubation with dsDNA, ssDNA, and ssRNA. The autocorrelation curves were fitted with a one-component normal diffusion model (*i* = 1 in Eq. S1). The results are given in Table 1. The clear decrease in diffusion coefficient and increase in hydrodynamic radius was observed only in samples containing dsDNA (but not RNA or ssDNA).

**Table 1:** Diffusion coefficients and hydrodynamic radii were obtained for free indigo carmine dye, DNA-enriched IC solution, and RNA-enriched solution (the RNA radius ≈ 57 nm (Karpińska et al. 2021), the DNA radius ≈ 1 nm)

Probe	Diffusion coefficient (µm^2^/s)	Hydrodynamic radius (nm)
IC	475.20 ± 42.87	0.51 ± 0.05
IC + dsDNA	304.08 ± 30.21	0.79 ± 0.08
IC + RNA	445.32 ± 47.78	0.54 ± 0.06
IC + ssDNA	476.39 ± 68.61	0.50 ± 0.07

### IC protects bacteria against phage infections

**Fig. 4:**
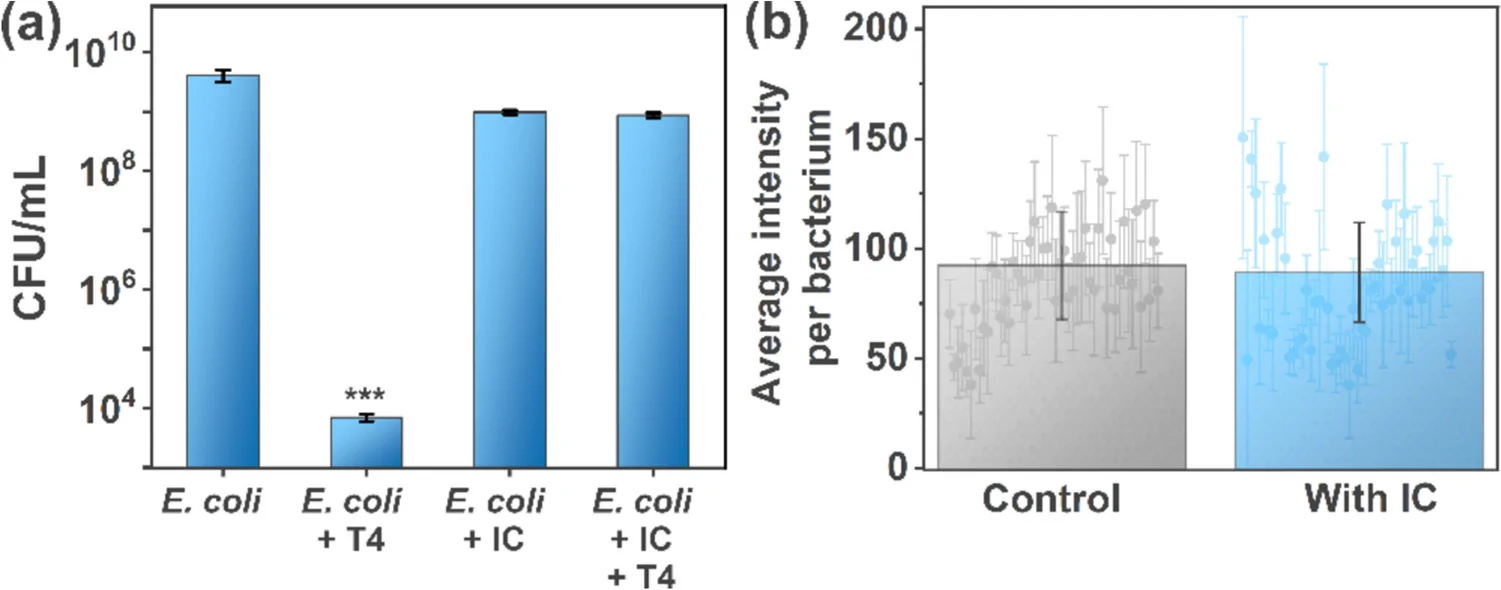
**a** Establishing the potential of IC as an antiphage agent by letting *E. coli* grow in the presence of both IC and T4 bacteriophages at 37 °C for 6 h. The panel shows the individual effects of IC (C) and T4 (B) on *E. coli*. IC protects *E. coli* from T4 infection (bar D). **b** A comparison of GFP expression in *E. coli* in the absence and presence of indigo carmine (IC) to determine the possible effects of IC on protein expression by bacterial cells. There were no statistically significant differences between the samples

The collected results allowed us to protect bacteria cultures against phage infections. The experiments were executed in optimal conditions for bacteria growth, i.e., in LB medium, 37 °C, and at 220 rpm. We analyzed the number of viable bacteria in the LB medium and LB medium containing IC. Next, we spiked the samples with the same number of T4 (final concentration of 10^3^ PFU/mL). The addition of IC resulted in a small decrease in the concentration of bacteria (from around 4 × 10^9^ CFU/mL to 9.8 × 10^8^ CFU/mL). The number of bacterial cells decreased upon spiking the culture with T4 phages by around 6 log (from around 4 × 10^9^ to 7 × 10^3^ CFU/mL). However, when IC was present in the medium before the addition of T4, *E. coli* was protected. There was no statistically significant difference in the number of viable *E. coli* cells between samples containing IC and IC along with T4 (*P* > 0.05). The results are presented in Fig. 4a.

Figure 4b shows a comparative study of green fluorescent protein (GFP) induction in *E. coli* in the absence (control) and presence of indigo carmine. It was observed that the addition of IC to an *E. coli* culture did not impact the overexpression of the example protein (GFP), as the overall production of GFP was comparable to that of the control (without IC).

## Discussion

This study explores the novel use of indigo carmine (IC) as a selective and effective antiphage agent., particularly against DNA phages. IC inactivates tailed DNA phages by interacting with their dsDNA, leading to capsid deformation and genome ejection, as observed in fluorescence correlation spectroscopy and TEM imaging. The observed selectivity arises from IC’s inability to bind RNA, as demonstrated in experiments with MS2, the only RNA phage tested.

Temperature and agitation significantly enhance IC’s efficacy, achieving a 7-log reduction in T4 phage titer at 50 °C under agitation (220 rpm), while lower temperatures and static conditions require higher IC concentrations (Fig. 1b). These findings align with kinetic theory, suggesting mechanical mixing increases collision frequency, facilitating IC-phage interactions.

IC offers a cost-effective, environmentally safe, and FDA-approved alternative compared to existing methods, such as toxic chemical agents and expensive nanoparticle-based strategies. Unlike quaternary ammonium compounds or gold nanoparticles (Hora et al. 2020), IC does not harm bacterial growth or protein expression, as confirmed in *E. coli* cultures with GFP production. The observed compatibility with industrial processes makes IC a strong candidate for protecting bacterial cultures from phage contamination.

Mixing facilitates transport, thereby influencing the number of collisions between IC molecules and virions. However, the changes in the studied range of temperatures had a rather complex effect on the movement of the system’s constituents. Our recent study reveals that the presence of indigo carmine in an aqueous solution lowers the temperature at which slowed motion of the solution components occurs, with temperature-dependent changes in the water’s local structure being a key factor (Atamas et al. 2024). We showed that the diffusion coefficient of water in the IC-water system increased with the temperature, whereas the diffusion of IC itself decreased (cf. Figure 4 in (Atamas et al. 2024)). The previously published neutron scattering data demonstrated the changes in the movement of water molecules in the physiological temperature range and indicated a correlation between the effect of lipid hydration and water dynamics (Swenson et al. 2008). The experimental dependence between the antiphagent activity of IC and temperature in the range from around 25 to 50 °C resembled the temperature dependence of the diffusion coefficient of water in the system. Alterations in the properties of water itself in the physiological temperature range might lead to a change in the interactions between the components of the systems (Atamas et al. 2021).

We also analyzed the possibility that the permeability of the capsid increased at higher temperatures, allowing for easier entry of indigo carmine molecules (Serwer & Wright 2018). We observed that the inactivation of bacteriophages occurred only when virions were incubated with indigo carmine at an elevated temperature This suggested a time-dependent mechanism that most likely relied on the movement of IC molecules. We hypothesize that this mechanism might be related to the movement of IC molecules from the solution through the capsid toward the genome inside (Winogradoff et al. 2022).

To verify the versatility of IC as an antiphagent, we studied six different phages: T1, T4, T7, MS2, λ, and P001. T1, λ, and T7 phages are non-enveloped and have a simple head–tail structure, wherein the tail is non-contractile (Hyman & Abedon 2009; Pell et al. 2009). T4 phages belong to the most abundant family, with a prolate head and a contractile tail (Miller et al. 2003). *Lactococcus* phage (P001) belongs to *Siphoviridae*, and it is infamous for disrupting dairy fermentation processes (Chmielewska-jeznach et al. 2018). We chose this phage to create a model for industrial applications. All studied tailed phages store their genetic material in the form of dsDNA. MS2 belongs to *Leviviricetes*, consisting of a capsid in which genetic material (RNA) is stored*.* MS2 is regarded as an excellent model for researching viruses that infect eukaryotic cells (Raza et al. 2023). It was used as a model to study eukaryotic viruses like COVID-19 (Brady 2024; Desai et al. 2023), human norovirus (Shaffer et al. 2024), and other enteric viruses (da Silva et al. 2023). It is also a challenging target, as it can withstand the action of agents deactivating other phages (Richter et al. 2021a, b). We tested the inactivation rate for all six studied phages upon incubation with IC at a broad range of concentrations. This data was fitted using the Hill equation to establish EC_50_, i.e., the half-maximal effective concentration. At 50 °C, EC_50_ varied from 0.105 to 0.006 mg/mL for DNA phages (T4 and λ, respectively; Fig. 3a–e). We found that IC caused the contraction of the sheath and the ejection of DNA from the capsid in the case of T4 (Fig. 2). No activity against MS2 phage, i.e., RNA phage, was found even at high IC concentrations.

The apparent selectivity of IC underlined the importance of interactions with the genetic material. FCS studies were performed to check whether indigo carmine molecules bind to dsDNA, ssDNA, and RNA (Fig. S3 a-d). The following options were possible: (1) the dye does not bind to nucleic acid strands, and no change in the diffusion coefficient should be observed, and (2) the dye binds to nucleic acid strands, which would decrease the diffusion coefficient of the dye. Analysis of the FCS data showed that in the system containing dsDNA, the diffusion coefficient values were much lower (approximately 50%, cf. Table 1) than in all other studied systems (i.e., only IC, IC + RNA, and IC + ssDNA). This suggested that dsDNA moved together with IC. The diffusion coefficients of IC measured in systems containing ssDNA and RNA were within the calculation error limit—the determining factor that affected the movement in these systems is the movement of indigo carmine and its interaction with the environment, including water, rather than interaction with the ssDNA or RNA molecule.

TEM pictures of inactive T4 bacteriophages indicated a tail contraction and DNA release (Fig. 2). T4 bacteriophages utilize contractile injection of their genome into *E. coli*, assuming a “contraction wave” pathway (Maghsoodi et al. 2019). When T4 comes in contact with the *E. coli* host cell, the tail fibers bind to the outer membrane of the host, which results in conformational changes in the geometry of the baseplate (Wenzel et al. 2020). As a result, the tail contracts, while the tail tube is believed to puncture the bacterial cell envelope mechanically (Maghsoodi et al. 2019). Tail sheath contraction can otherwise be initiated artificially by changing the pH or using chemical agents like cationic detergents or urea (Aksyuk et al. 2009). Contraction of the tail is an example of artificial shrinking in the presence of an external agent, i.e., IC. Due to this contraction, bacteriophages receive a signal to eject DNA.

NMR and HPLC analysis eliminated the possible impact of impurities in quantities that can bring about the effect. A dye sample dissolved in D_2_O (*c* = ca. 10 mg/mL, i.e., much higher than used in the experiments) was subjected to ^1^H NMR analysis (Fig. S4a). The spectrum showed tree peaks attributed to the protons in the aromatic ring of the dye (the N–H proton was not recorded due to the fast exchange of this atom for deuterium). Apart from the prominent peaks, signals of the impurities were found in the 8.2–6.6 ppm region. The sample was then incubated at 50 °C for over 96 h, and the spectrum was collected again. No new peaks were present, and the ratio of peaks’ integrals to the impurities’ signals did not change. This result suggested that no new compounds other than those already present in the starting material were created upon incubation. The ratio between surface areas under the peaks of protons attributed to the dye and the impurities changed from 15:1 to 13:1. This indicates a slight compound degradation during incubation. To further confirm this observation, samples were subjected to HPLC analysis (Fig. S4b). The chromatograph showed the presence of three compounds at 0.743, 0.933, and 2.724 min, accordingly. The second signal can be attributed to IC dye (Violeta & Trandafir 2007), and the remaining signals are probably of mono- and desulfonated compounds of IC (De Keijzer et al. 2012). The NMR and HPLC data are provided in the Supporting Information.

To validate the efficacy of IC (food dye), we tested indigo carmine purchased from Sigma-Aldrich (Merck). Both compounds have the same molecular structure. We observed a significant reduction in phage titer (> 99.9%) at 0.5 mg/mL under optimized conditions (Fig. S5), highlighting its strong antiphage activity comparable to commercially available food-grade indigo carmine.

This study introduces the novel concept of utilizing indigo carmine (IC), a common food additive, as an effective and selective antiphage agent for industrial applications. The innovation lies in its ability to selectively inactivate DNA phages through direct interaction with their genetic material while exhibiting no activity against RNA phages. This selectivity was confirmed through fluorescence correlation spectroscopy, which demonstrated a significant binding affinity of IC to dsDNA but not to RNA or ssDNA. Mechanistically, IC permeates the capsid, induces capsid deformation, and facilitates genome ejection, as validated by transmission electron microscopy.

The findings highlight that under optimized conditions—50 °C with mechanical agitation (220 rpm)—IC achieved complete inactivation of phages, reducing phage titers by up to 7 logs. Remarkably, IC demonstrated no adverse effects on bacterial growth or protein production, as evidenced by GFP expression assays. Its compatibility with bacterial processes and its FDA approval, environmental safety, and cost-effectiveness underscore its potential as a scalable solution for safeguarding industrial bacterial cultures.

Compared to existing methods, such as harsh chemical disinfectants and radiation-based strategies, IC offers a safer and more targeted approach without compromising bacterial viability or product quality. For instance, previous studies on gold nanoparticles have shown antiphage properties but come with prohibitive costs and complex preparation methods. IC stands out as a readily available, cost-effective alternative that meets the needs of industrial-scale operations.

In conclusion, this study establishes indigo carmine as a pioneering antiphage agent that bridges the gap between efficacy, safety, and scalability. Future research should focus on enhancing IC’s efficiency against RNA phages and exploring structural modifications to broaden its applicability. Furthermore, future studies could focus on developing methods to remove dyes from water, building on existing research, to mitigate environmental contamination effectively (Rehan et al. 2023; Waliullah et al. 2023). The findings open new avenues for the development of antiphage strategies that are sustainable, efficient, and compatible with diverse industrial bioprocesses.

## Supplementary Information

Below is the link to the electronic supplementary material.

Supplementary file1 (PDF 527 KB)

## Data Availability

The raw data required to reproduce these findings are available to download from https://doi.org/10.18150/AGRBJL.
