# Supplementary material for: The activity of indigo carmine against bacteriophages: an edible antiphage agent
Source: Appl Microbiol Biotechnol. 2025 Jan 25;109:24. doi: 10.1007/s00253-025-13414-4 (PMC11762416; doi:10.1007/s00253-025-13414-4)
Supplement: Supplementary file 1 [file 253_2025_13414_MOESM1_ESM.pdf]

Supporting Information for

The activity of indigo carmine against bacteriophages - an edible antiphage agent

Sada Raza[1], Bartłomiej Bończak[1], Nataliia Atamas[1,2], Aneta Karpińska[1], Tomasz Ratajczyk,[1] Marcin Łoś[2,3], Robert Hołyst[1], Jan Paczesny[1,*]

Corresponding author: Jan Paczesny, Ph.D., +48 22 343 2071

Email: jpaczesny@ichf.edu.pl

Materials and Methods:

Fluorescence Correlation Spectroscopy (FCS)

Probes used: dsDNA solution (double stranded, 69 bp per molecule, solution concentration = 20 nM, sequence = GAT ACG AGC ATC GTG TAG GCA TCG TAG GTA ATA CGG ATG TGC TAG CTT ATT GAA TTC AGA GAT CTA TGC), ssDNA solution (single stranded, 69 bp per molecule, solution concentration = 20 nM, sequence = GCA TAG ATC TCT GAA TTC AAT AAG CTA GCA CAT CCG TAT TAC CTA CGA TGC CTA CAC GAT GCT CGT ATC), indigo carmine (concentration = 100 nM). The affinity of IC to RNA was verified using RNA control 250, catalog number # AM7155 (Thermo Fischer Scientific). The concentration of RNA used in FCS measurements = 10 nM. The studies were conducted in PBS solution.

Data were fitted using the normal diffusion model, **Equation S1**:

$$G(\tau) = \frac{1}{N}\left(1 + \frac{\theta_{trip}}{1-\theta_{trip}}\exp\left(-\frac{\tau}{\tau_{trip}}\right)\right)\sum_{i=1}^{n} A_i \frac{1}{1+\left(\frac{\tau}{\tau_{Di}}\right)}\frac{1}{\sqrt{1+\frac{1}{\kappa^2}\left(\frac{\tau}{\tau_{Di}}\right)}} \quad \textbf{Equation (S1)}$$

where N stands for overall particle number in confocal volume, θ_{trip} – a fraction of particles in a triplet state, τ_{Di} corresponds to the diffusion time of the i-th diffusing component, Ai is the amplitude of the i-th component, κ stands for the aspect ratio of the Gaussian used to approximate the focus. During calibration, the parameter κ was determined and was in the range of 5.5-6.0. All FCS data included in the manuscript were fitted with a one-component normal diffusion model.

Nuclear magnetic resonance (NMR)

Proton NMR spectra were recorded on an Agilent DD2 400MHz spectrometer (operated with Open VNMRJ 2.1 DDR software) in deuterated solvents at the temperature of 297 K. Proton chemical shifts are reported in ppm (δ) relative to tetramethyl silane (TMS) with the solvent resonance employed as the internal standard (D_2O δ 4.79 ppm; DMSO-D_6 δ 2.50 ppm). The solvents were purchased from ROTH and used without purification.

High-performance liquid chromatography (HPLC)

HPLC analysis was performed on Chromatograph Acquity UPLC Waters with BEH C18 column, 17 μm 2.1 mm x 100 mm. A mixture of 100 mM ammonium acetate pH=7 and acetonitrile (9:1, v/v) was used as the mobile phase, with a 0.3 mL/min flow. The column temperature was maintained at 35 °C. A diode array detector (DAD) was used to register the chromatogram with a wavelength of 608 nm. The sample size was 1 μL, with a 0.25 mg/mL concentration.

Results

An experiment was conducted to record the change in PFU/mL of T4 bacteriophages as the concentration of indigo carmine varied from 0 mg/mL to 4 mg/mL. The bacteriophages were incubated at 37 °C for 24 hours for each concentration, followed by titration. It was observed that lower concentrations, such as 0.5 mg/mL and 1 mg/mL, did not significantly affect the inactivation of bacteriophages at 37°C. However, at a concentration of 1.5 mg/mL, an inactivation of approximately 0.5 log PFU/mL was visible. Further increases in concentration, from 2 mg/mL to 4 mg/mL, resulted in a consistent inactivation level, with no further reduction beyond 0.8 log PFU/mL**.**

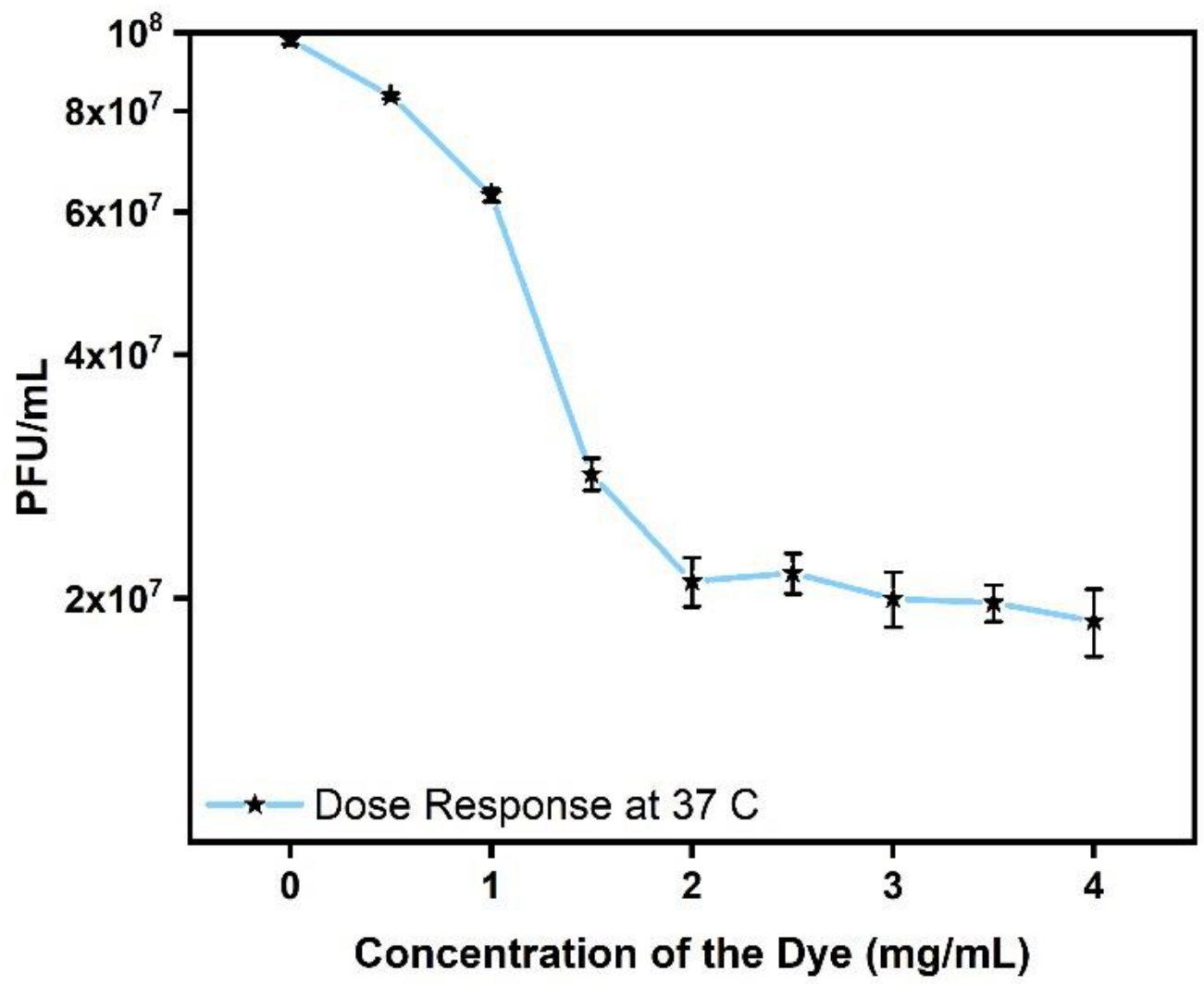

Figure S1. Change in PFU/mL of T4 bacteriophages as the concentration of indigo carmine varies from 0 mg/mL to 4 mg/mL. The bacteriophages were incubated at 37 °C for 24 hours for each concentration, followed by titration.

The next experiment was designed to establish the root cause of inactivation. Five different samples were incubated at 50 °C and titrated as follows: A) a control sample with only phages in TM buffer (no indigo carmine), B) indigo carmine (IC) only incubation followed by the addition of phages, C) phage-only incubation followed by the addition of IC just before titration, D) co-incubation of IC and phages together followed by titration and E) separate incubation of IC and phages followed by their mixing and immediate

titration. It was found that a reduction in PFU/mL of T4 bacteriophages was only observed in the case of co-incubation of IC and T4. In other cases, where IC and T4 were incubated separately at elevated temperatures and mixed just before titration, phages were not inactivated. Also, incubation of only IC or only T4 did not lead to the consequent inactivation either.

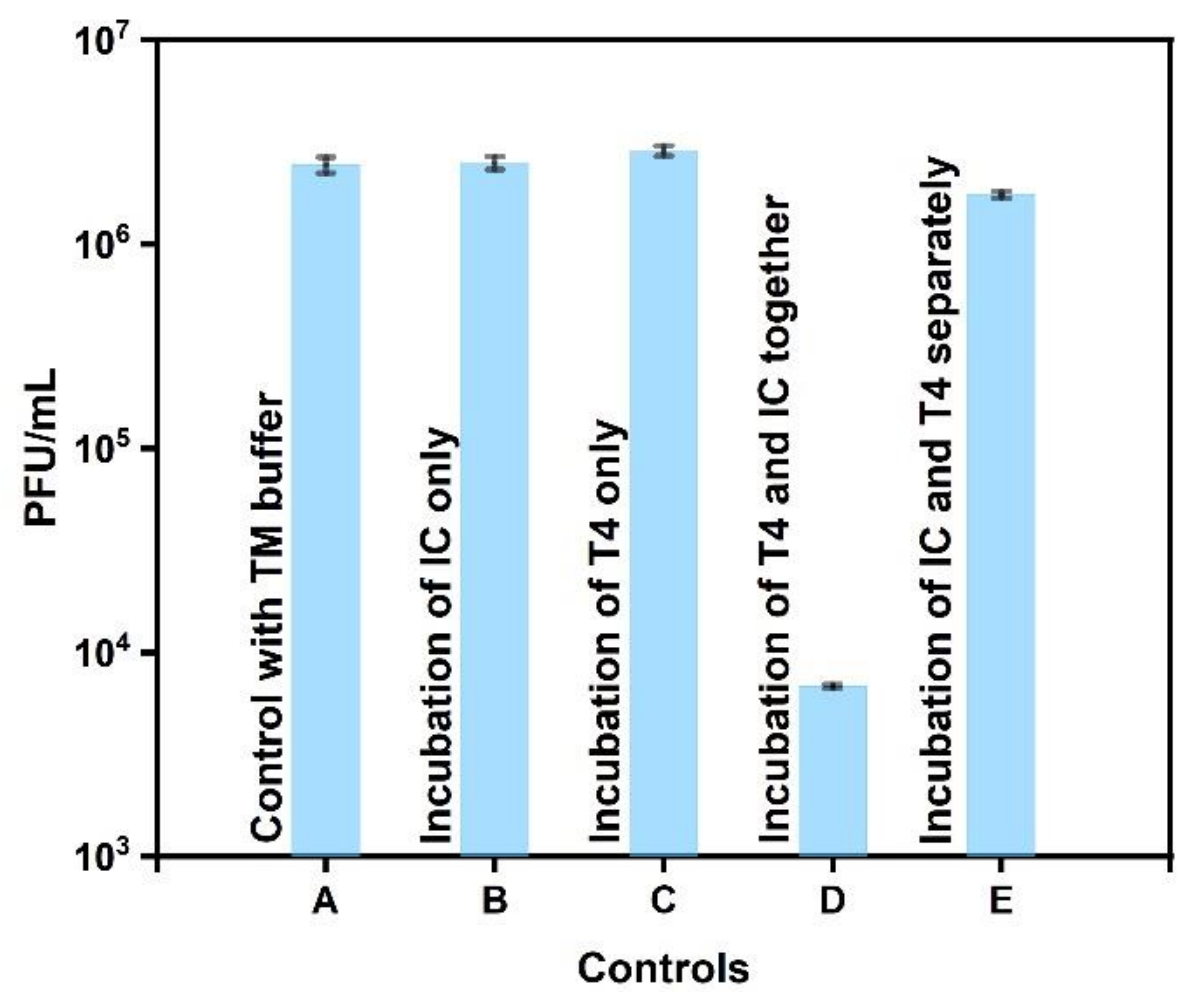

Figure S2. The panel shows measurements of titer of T4 phage suspension to establish the root cause of inactivation. Five different samples (left to right) were incubated at 50 °C and titrated as follows: A) control sample with only phages in TM buffer (no IC), B) IC only incubation followed by addition of phages, C) phage only incubation followed by addition of IC just before titration, D) incubation of IC and phages together followed by titration and E) separate incubation of IC and phages followed by their mixing and immediate titration.

FCS (Fluorescence Correlation Spectroscopy) studies were performed to determine whether indigo carmine molecules bind to dsDNA, ssDNA, and RNA. Two potential outcomes were considered: 1) the dye does not bind to nucleic acid strands, resulting in no change in the diffusion coefficient, and 2) the dye binds to nucleic acid strands, which would decrease the diffusion coefficient of the dye. Analysis of the FCS data revealed that in the system containing dsDNA, the diffusion coefficient values were significantly lower (approximately 50%, as shown in Table 1) compared to other studied systems (only IC, IC+RNA, and IC+ssDNA). This indicated that dsDNA moved together with IC. In contrast, the diffusion coefficients of IC in systems containing ssDNA and RNA were within the calculation error limit, suggesting that the movement in these systems was primarily influenced by the interaction of indigo carmine with the environment, including water, rather than with the ssDNA or RNA molecules.

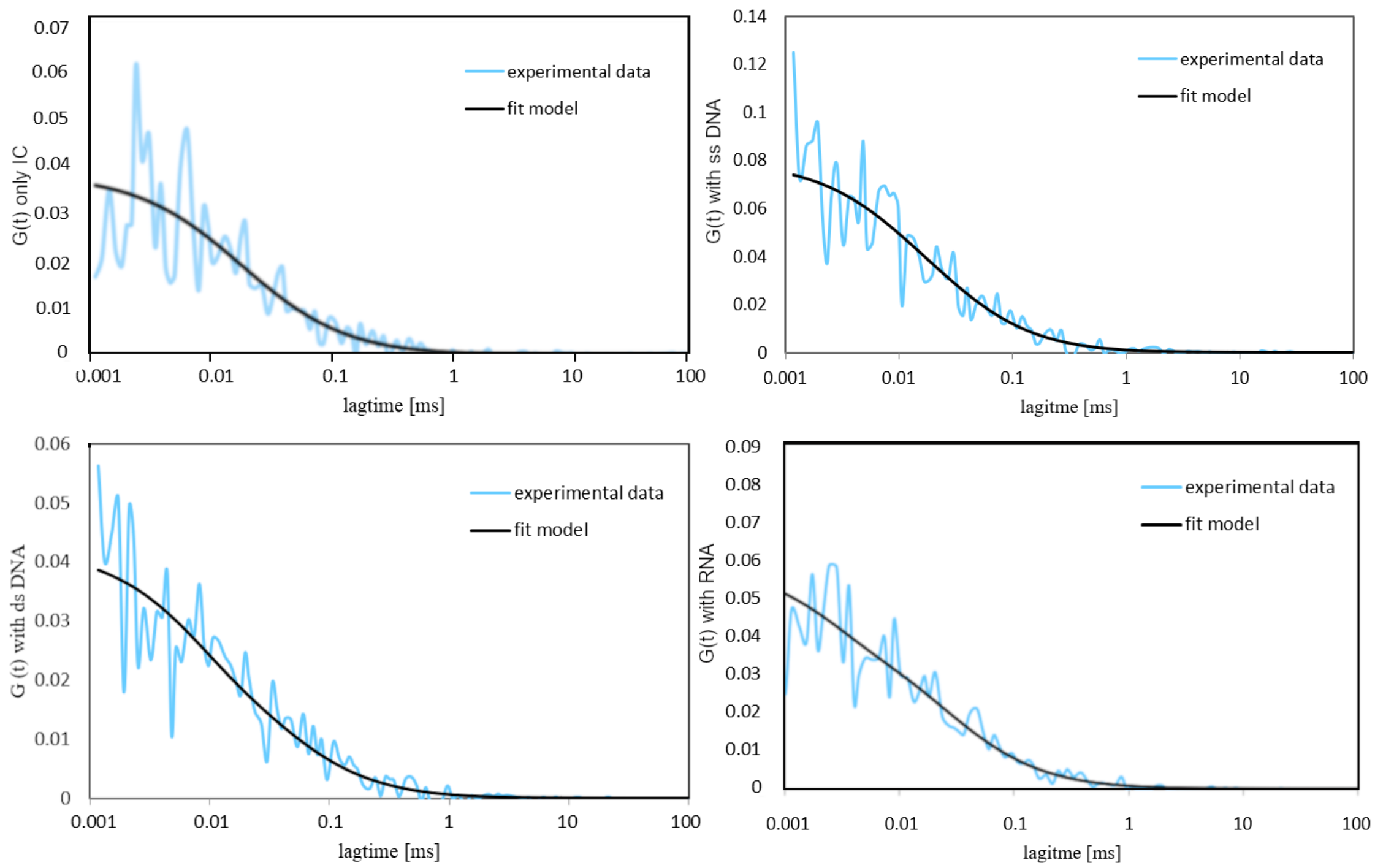

Figure S3. FCS autocorrelation curves fitted with a one-component normal diffusion model, displaying only IC **(a)**, IC with ss DNA **(b)**, IC with ds DNA **(c)**, and IC with ss RNA **(d)**.

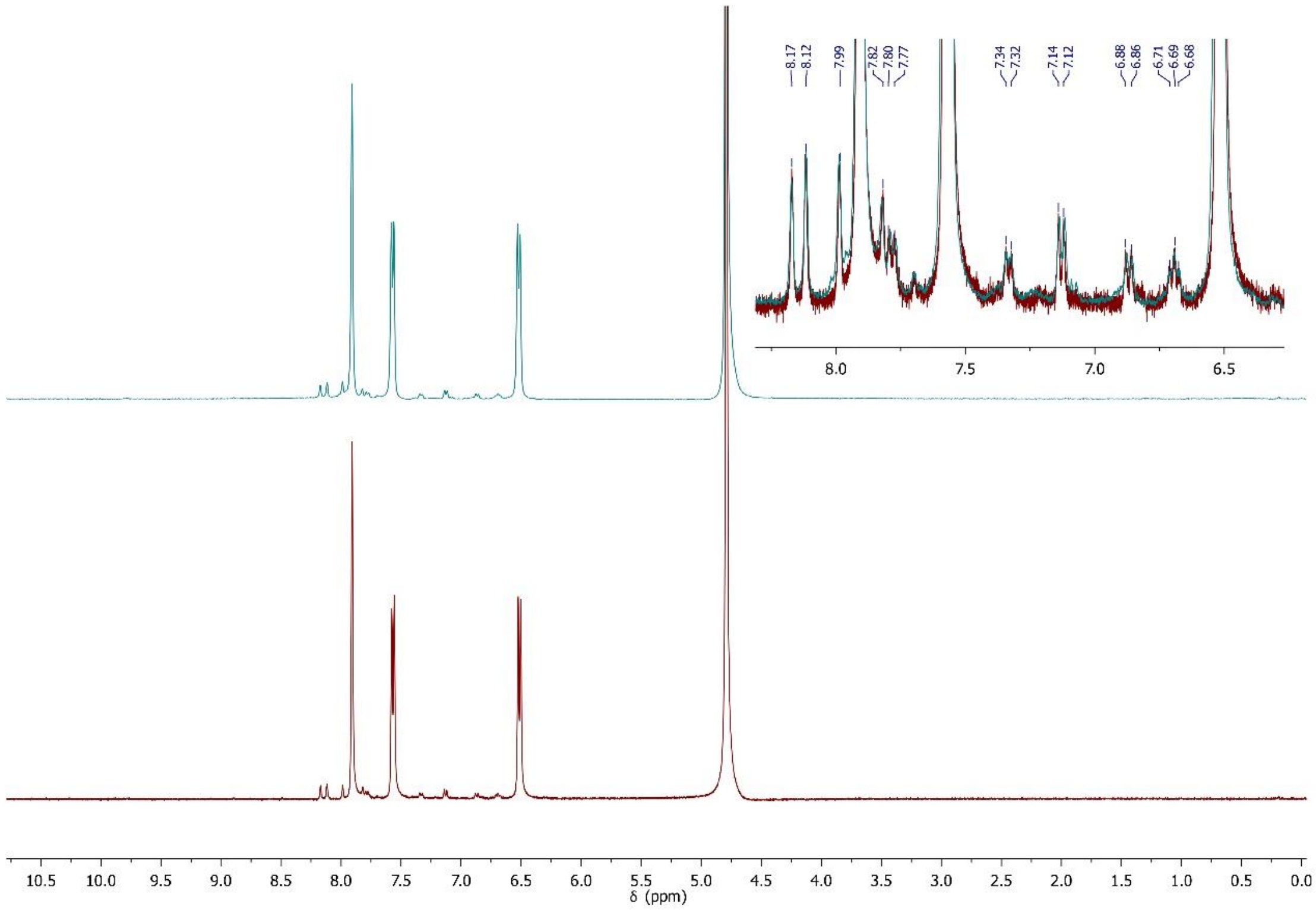

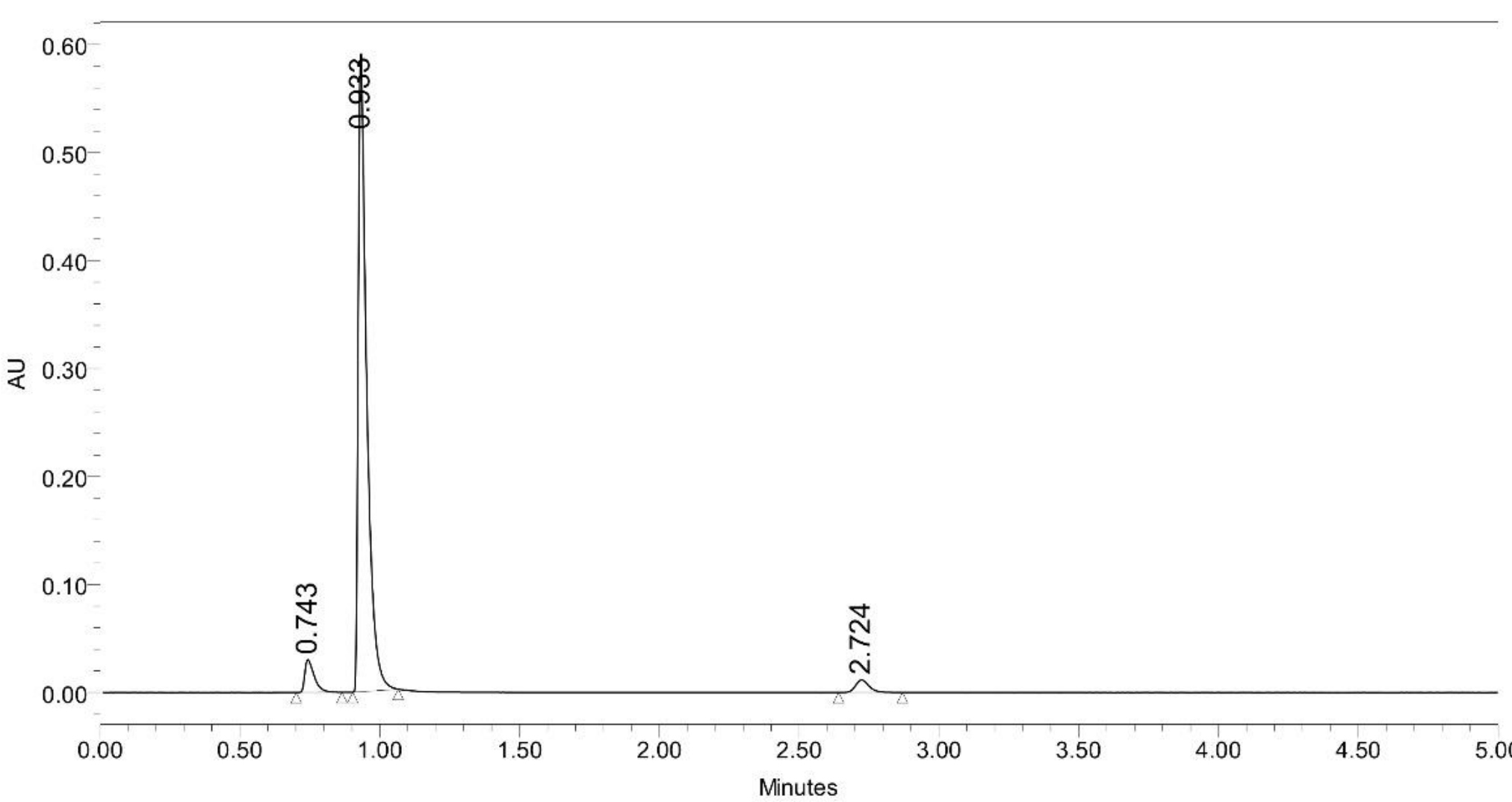

	RT	Area	% Area	Height	Width (sec)
1	0.743	70809	5.20	30270	9.800
2	0.933	1251027	91.91	591399	9.800
3	2.724	39325	2.89	11651	13.700

Figure S4. (a) Comparison of ^{1}H NMR graphs of freshly prepared (red) and incubated (50 °C for 96 h; blue) solutions of Indigo Carmine in D_2O. **(b)** HPLC of Indigo Carmine dye.

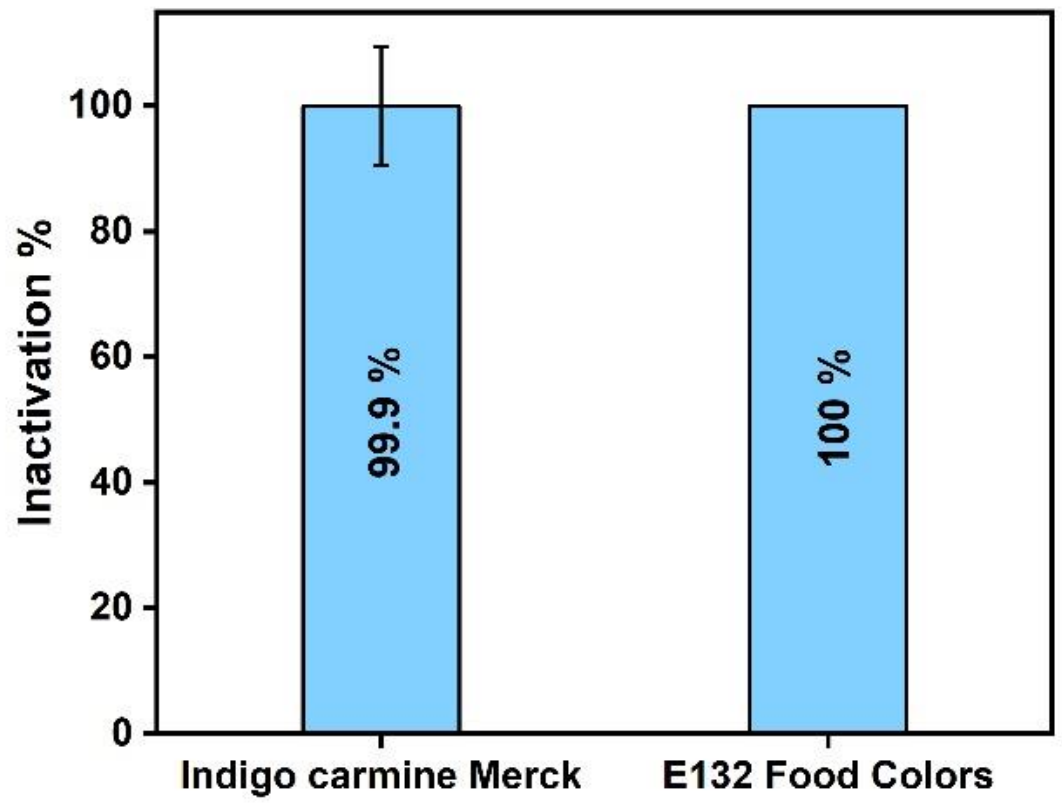

Figure S5. Effect of indigo carmine from Sigma-Aldrich (Merck) and E132 (obtained from Food Colors) on bacteriophage inactivation. The bar graph compares effect of indigo carmine from Merck and E132 from Food Colors at 0.5 mg/mL with 24 hours incubation at 50 °C.
